# Arthrogryposis multiplex congenita causing hypercapnic respiratory failure and airway obstruction in an adult patient

**DOI:** 10.1002/rcr2.1361

**Published:** 2024-04-23

**Authors:** Harshana Bandara, Sathiaruban Sathiyamoorthy, Dinusha Withana, Tharindi Dissanayake, Harshani Athapaththu, Wathsala Gunasinghe

**Affiliations:** ^1^ Northwest Lung Centre Wythenshawe Hospital Manchester UK; ^2^ Department of Respiratory Medicine National Hospital for Respiratory Diseases Welisara Sri Lanka; ^3^ Department of Internal Medicine Base Hospital Nikewaratiya Sri Lanka

**Keywords:** arthrogryposis multiplex congenita, hypercapnoiec respiratory failure, leak port, non‐invasive‐ventilation, trache‐ventilation

## Abstract

This case highlights arthrogryposis multiplex congenita (AMC) as a rare cause of hypercapnoeic respiratory failure and airway obstruction in adults and emphasizes the usage of leak‐port in tracheostomy‐NIV (non‐invasive‐ventilation) specially in resource poor setting when the tracheostomy‐NIV mask is unavailable.

## Graphical abstract

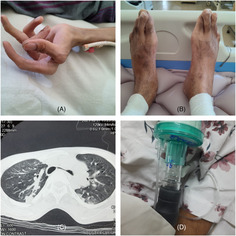

## CLINICAL IMAGE

**FIGURE 1:**
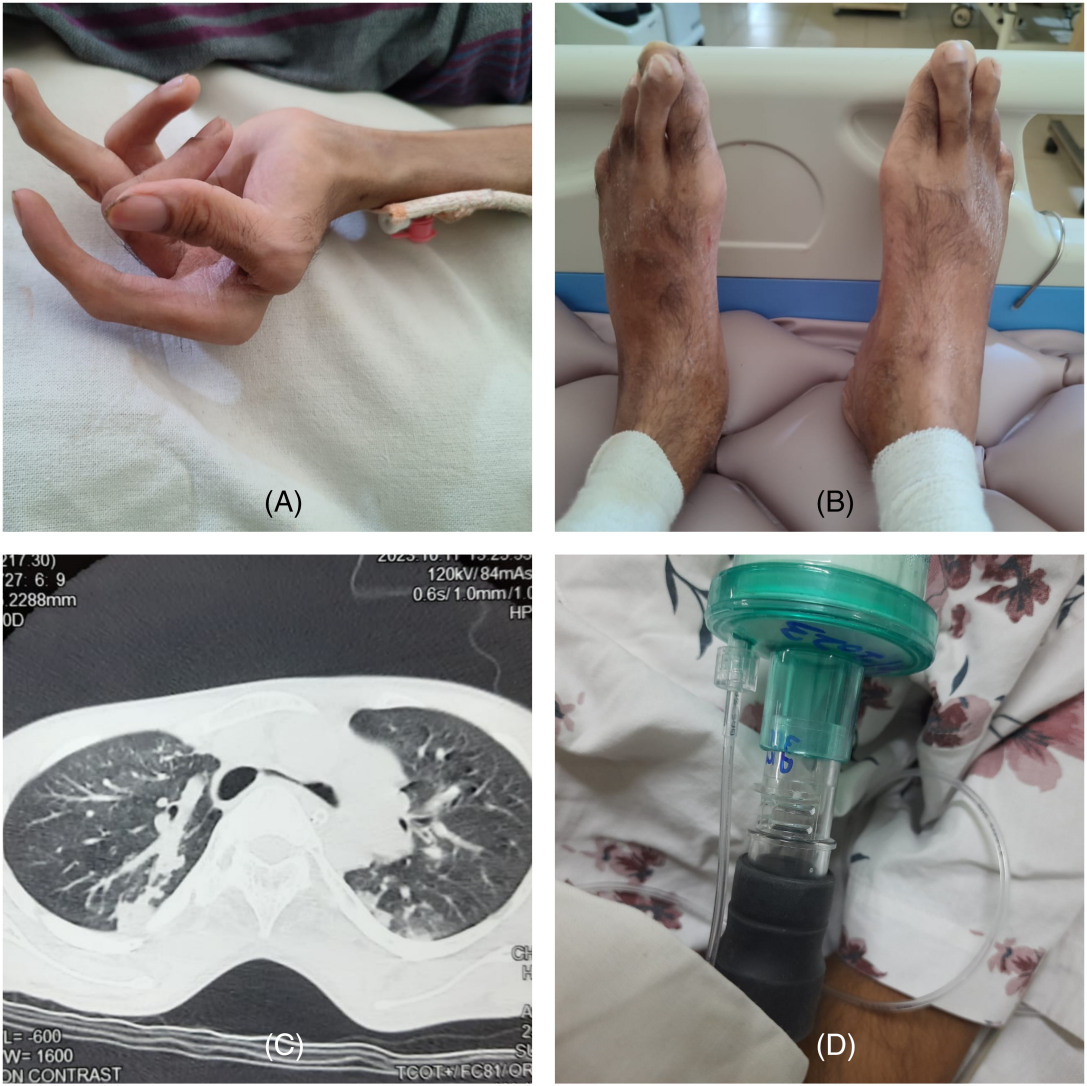
This illustrated the hand and the feet deformities secondary to arthrogryposis multiplex congenita (A, B). The computed tomography (CT) axial images showing the narrowing of the left main bronchus due to the reduction of antero‐posterior diameter of the chest wall causing the left main bronchus being sandwiched between the great vessels and the spine (C). The image illustrates the single limb non‐invasive‐ventilation connection through leak‐port and to the tracheostomy allowing the exhalation (D).

A 26‐year‐old gentleman, a non‐smoker presented with worsening dyspnoea and drowsiness. He had a neonatal diagnosis of Arthrogryposis multiplex congenita (AMC) with deformed hand‐foot joints (Figure 1A,B). No other significant comorbidity and he was fully independent despite deformities with very low BMI of 16 kg/m^2^. He had symptoms suggesting chest infection with raised inflammatory markers with chest x‐ray changes of chronically reduced left lung volume without major consolidations. Arterial‐blood‐gas showed acute‐on‐chronic hypercapnoeic‐respiratory‐failure (HRF). Previous blood‐gases suggested pre‐existing compensated HRF. He was intubated and ventilated and treated with antibiotics. Computer‐tomography showed bilateral aspiration pneumonia with narrowed left‐main bronchus being sandwiched between the great vessels and the spine due to his narrow antero‐posterior diameter secondary to chest‐wall joint deformity due to AMC (Figure 1C). He underwent tracheostomy due to the difficulty weaning on 14th day of intubation. He was weaned to single‐limb NIV via tracheostomy using the leak‐port for exhalation (Figure 1D). Later he was weaned to long‐term NIV via facemask while decannulating the tracheostomy.

AMC is a rare disorder of new‐borns causing congenital fixed joint contractures. Environmental and genetic causes have been discussed as potential aetiologies.^1^, ^2^ Limitations of the movements in foetal‐life, neuromuscular‐diseases, spina‐bifida, muscular‐dystrophy, and sometimes a maternal history of multiple‐sclerosis are also considered as the causes.^3^ As the joints in the chest wall also can be affected, new‐borns can go into respiratory failure. Airway obstruction and HRF in adults with AMC is a rare but important potentially complication that must be considered in respiratory presentations of these patients.

## AUTHOR CONTRIBUTIONS

Harshana Bandara conceptualized and wrote the first draft. All the authors revised subsequent versions and approved the final version.

## CONFLICT OF INTEREST STATEMENT

None declared.

## ETHICS STATEMENT

The authors declare that appropriate written informed consent was obtained for the publication of this manuscript and accompanying images.

## Data Availability

Data sharing not applicable to this article as no datasets were generated or analysed during the current study.
